# Antibiotic treatment of honey bee colonies alters early gut microbiome assembly and induces persistent dysbiosis in newly emerged workers

**DOI:** 10.1038/s41598-025-12823-9

**Published:** 2025-08-08

**Authors:** Nathan O. Allen, Duan C. Copeland, Brendon M. Mott, Robert Erickson, Kirk E. Anderson

**Affiliations:** 1 Entomology and Insect Science Graduate Interdisciplinary Program, University of Arizona, Tucson, AZ 85721 USA; 2 USDA-ARS Carl Hayden Bee Research Center, 2000 E. Allen Rd, Tucson, AZ 85719 USA

**Keywords:** Microbiota, Antibiotics, Honey bee, Colony-level, Antibiotic resistance, Tylosin, Gut microbiome assembly, Antimicrobials, Microbial communities

## Abstract

The honey bee worker gut microbiome is assembled during the first days of adult life and, within the first week, matures to a relatively stable state that contributes to host health and behavior. Species composition, spatial distribution in the gut, and temporal species succession patterns all follow predictable and consistent patterns, creating a recognizable healthy worker gut microbiome. Though these quantities change with the age, task, and diet of the host, the mature microbiome is robust to minor disturbances. Mechanisms driving healthy microbiome assembly remain unclear, but abiotic, host-microbe, and microbe-microbe interactions are likely important to this development. Worker microbiomes may be altered to a dysbiotic state through nutritional, pathogen, and antibiotic stressors, increasing individual and colony susceptibility to further injury. Antibiotic use for control of bacterial diseases of larvae has been common beekeeping practice for decades, however, negative effects on the gut microbiota have been shown to decrease survivorship of affected workers and alter task-related behavioral patterns. We examined the succession of the worker gut microbiome across the first three weeks of adulthood in bees treated with the common beekeeper antibiotic tylosin. We found that both microbiome size and structure were significantly altered by tylosin treatment in 1 day old bees, and these effects persisted more than 2 weeks after last treatment application and did not recover to match control microbiomes by 21 days and the time of typical foraging onset. Certain *Bifidobacterium and Bombilactobacillus* species were strongly depleted by treatment, creating persistent dysbiotic states. These results illustrate early microbiome assembly in the worker gut and the negative effects of tylosin treatment on dynamic microbiome maturation.

**Supplementary Information:**

The online version contains supplementary material available at 10.1038/s41598-025-12823-9.

## Introduction

Animal gut microbiomes impact the health and life history of their hosts through effects on nutrition and immunity, and even modulate host development and behavior^1^. In honey bees, the gut microbiome is essential to worker health and immunity, and may even be an important determinant of behavior and learning^2–4^. Dysbiotic bees with atypical microbiome abundance and composition are more susceptible to infection and even show higher mortality in the absence of infection^5–8^.

The healthy worker gut microbiome of honey bees is highly consistent in composition, with five core bacterial lineages found in all workers. These lineages consist of species within the genera *Snodgrassella*, *Gilliamella*, *Bifidobacteria*, and two genera of *Lactobacillaceae: Lactobacillus* and *Bombilactobacillus*, formerly known as Firm-5 and Firm-4 respectively^2^. Closely related to *Gilliamella*,* Frischella* is also prevalent and abundant, and often considered core^9^. Four colony co-evolved bacterial lineages are found more sporadically in worker guts: *Apilactobacillus* and *Bombella* are associated with the colony social resource niche, which includes both stored and worker-secreted nutritional resources and developing larvae^10^. The genera *Bartonella* and *Commensalibacter* also occur sporadically, seasonally, or with increased age in worker guts^11,12^.

The bacteria that comprise the gut microbiome exist in a complex network of competitive and synergistic interactions, where each species may perform multiple roles, acting as a collaborator and competitor to numerous other species at once. This network takes time to establish and changes over the course of a worker’s life, in response to changing diet, task, and immune challenge^2,13,14^. Immediately after adult emergence, newly emerged workers (NEWs) have little to no bacterial abundance in the gut, but through exposure to nestmates, nutrition and colony materials, the microbiota quickly develops^9,14,15^. The initial acquisition of all core genera happens in the first 4–7 days following adult emergence, but a dynamic succession of increasing species and strain diversity continues throughout the life of a worker^9,15^.

Species of the core microbiome exhibit correlated patterns of co-occurrence and total abundance, and partner choice between microbiome members may impact the functional capacities of the microbiota as a whole^13^. These correlations extend to the level of bacterial strain, as strains of many of the core microbiome species coexist within colonies but show a pattern of single-strain dominance in individual bees^16^. These strains can vary significantly in functional gene content, with consequences for their interaction with the rest of the microbiota and the host^17,18^. When and whether strains or species among the core microbiome members establish can depend on nutritional factors, exposure to nestmates, the host genetic background, direct microbe-microbe interactions, phage predation, and perturbation by pesticide or biocide^13,19–23^. Disturbances to the worker gut microbiome are associated with a reduction in pathogen resistance and longevity, and with behavioral changes^5,24^. Microbiomes may persist in dysbiotic states permanently after disturbances, with negative impacts on host function^8^.

One such disturbance with practical impact on beekeeping is antibiotic use. Decades of oxytetracycline and tylosin application in beekeeping have made resistance widespread among core microbiome species. The strength of resistance varies among species and within species, with strains of *S. alvi* and *Gilliamella* spp. showing large differences^6^. Of the two major veterinary antibiotics, tylosin has been approved for use in beekeeping more recently and remains effective against some oxytetracycline-resistant strains of *Paenibacillus larvae*, the cause of the devastating larval disease American Foulbrood^25–27^. Tylosin persists in honey and may continue to influence the abundance and composition of colony microbial populations for months after initial treatment^28^.

Tylosin targets primarily Gram-positive bacteria, but affects some Gram-negative species as well. Since the honey bee gut microbiota consists of both Gram-positive and Gram-negative core members, with species and strain-level variations in antibiotic resistance, we expect unequal impacts of treatment on the community. This disproportionate effect on some members of the gut community may then disturb mutualist interactions or allow opportunist patterns of overgrowth among less affected taxa. Antibiotic treatments select for the most resistant species and strains within the colony, and we hypothesize that this has important impacts on microbiome assembly and dynamics through limiting availability of species and strains at the onset of gut microbiota colonization. The presence and traits of the earliest gut colonizers in newly emerged bees have a major impact on the time-sensitive succession of the gut microbiome, and some core species that establish later may have preferred microbial partners important to their establishment^3,13,15,29^.

In a previous experiment, we found that abundances of *Bifidobacterium* and *Bombilactobacillus* were depleted in putatively young bees collected after field tylosin application, and these colony-level effects persisted at least 33 days after treatment ended^10^. These worker bees were collected following a “fly-off” assay, suggesting they lacked flight experience and could be classified as young nurse-age bees. Another field study found reduced abundances of *S. alvi* and *Bifidobacterium spp.,* and an increase in susceptibility to pathogen infection among workers from tylosin-treated colonies^6^. This previous work revealed snapshots of how tylosin-disturbed microbiomes are structured, but only in bee samples of unknown age. In the present study, we examine the effects of antibiotic treatment on the assembly and dynamics of the worker gut microbiome with reference to worker age during ecological succession. We used 16S rRNA amplicon sequencing to determine the effects of tylosin on the initial community assembly and subsequent dynamics of hindgut microbiomes of newly emerged worker bees throughout the first 21 days of adult life.

## Methods

### Known-age worker cohorts

To track age within the colony environment, we marked newly emerged adult workers with a dot of paint on their thorax. To control for effects of host genetics on microbiome acquisition, capped brood frames from 12 different colonies were removed, brushed clean of bees, and placed in an incubator (35 °C, 95% humidity). After 24 h, newly emerged workers (NEWs) were brushed off brood frames into a bin, then hand painted with a dot of Posca water-based paint. Painted bees were divided into four equal groups of about 750 bees and placed onto the top bars of the bottom box of two experimental colonies. Each group of workers was lightly misted with warm 50% sugar syrup to encourage grooming and acceptance by new nestmates. Capped brood frames were maintained in incubators for 3 days and a cohort of NEWs was collected each morning, each distinguished by a different color of paint. This process created three known-age cohorts, each separated in age by 24 h.

At the Carl Hayden Bee Research Center apiary in Tucson, AZ, beginning on June 25, 2023, we chose two healthy colonies of approximately equal population and assigned them to a treatment, with or without antibiotic. To simulate conditions experienced by a NEW eclosing from a brood cell into a colony environment containing tylosin, colonies in the antibiotic group received a manufacturer’s recommended dose of tylosin powder. Treatment consisted of three 200-mg applications of tylosin mixed with powdered sugar, dusted over the cluster. Control hives were dusted with powdered sugar. This treatment was applied three times, reapplied every third day after the first application. NEWs of cohorts that were added on the same day as treatments were placed in the colonies about an hour after the first application of tylosin powder.

To collect workers following treatments, frames were briefly removed and we sampled 16 bees belonging to each age cohort. Bees were collected every third day for 7 sample collection timepoints, such that the cohorts of bees had been in the colony for 1, 2, and 3 days respectively on the first sampling day (see supplemental table S0 for visual diagram). The next sampling day was three days later such that the three cohorts were now aged 4, 5, and 6 days. This was repeated until it was impossible to recover 16 bees per cohort, when the oldest bees were 21 days old. Samples were immediately frozen on dry ice then stored in a −80 C freezer pending dissection and DNA extraction.

Frozen bees were thawed, then dissected to obtain hindguts. We dissected the hindguts into 2-ml bead-beating tubes containing 0.2 g of 0.1-mm silica beads and 300 µl of 1X TE buffer. Samples were bead beaten and homogenized for 2 min at 30-s intervals. To each sample, 100 µl lysis buffer (20 mM Tris–HCl, 2 mM EDTA, 5% Triton X-100, 80 mg/ml lysozyme, pH 8.0) was added followed by incubation at 37 °C for 30 min. DNA was then purified using a Thermo Fisher Scientific GeneJet Genomic DNA Purification Kit according to the manufacturer’s instructions for gram-positive bacteria.

### Bioinformatics

We performed high-throughput sequencing of the 16S rRNA bacterial gene to determine the microbiome structure of individual hindguts (*n* = 266). Sequencing was performed in two parts. Sequencing Run 1 was completed in February 2022 at the University of Arizona Genetics Core (UAGC) and Sequencing Run 2 took place in December 2023 through AZENTA Life Sciences (https://www.azenta.com/). Each performed internal quality control including DNA quantification before library preparation and empty cells to identify contaminants. Sequencing was performed using MiSeq (v3 PE-300 kit) following the manufacturer’s DNA library preparation protocol for amplification of the V3–V4 region of the 16S rRNA gene. Raw sequence data were returned for downstream analysis.

We analyzed the two runs of sequences together as a total of 13,560,696 quality-trimmed reads (300 bp assembled) reads across 280 libraries (Supplementary table S1). Run 1 produced a total of 10,241,064 reads, with library sizes ranging from 19,304 to 153,940, with a median of 72,994 and average of 73,150. Run 2 produced a total of 3,319,632 reads, with library sizes ranging from 14,248 to 32,712 with a median of 23,287 and an average of 23,301. The raw reads were combined prior to downstream processing.

16S rRNA gene sequences were processed using MOTHUR v.1.44.344^30^. Paired end reads were joined using the make.contigs command. After the reads were joined, we removed the first and last five nucleotides using the SED command in UNIX. Sequences were screened to remove ambiguous bases, using the screen.seqs command. Unique sequences were generated using the unique.seqs command. A count file containing group information was generated using the count.seqs command. Sequences were aligned to BEExact^31^ database using the align.seqs command. Sequences were filtered to remove overhangs and gaps using filter.seqs. The unique.seqs command was run again to remove new redundancies from filtering. A precluster step using pre.cluster was performed. Chimeras were removed using chimera.uchime command. Sequences that were not bacterial in origin were removed using the remove.seqs command. All unique sequences with only one or two reads (single/doubletons) were removed using the AWK command in UNIX. A distance matrix was constructed for the aligned sequences using the dist.seqs command. Unique OTUs were merged at the species-level with the merge.otus command. A raw total of 5179 unique OTUs were merged at the species level to obtain 236 species across all samples (Unique and merged sequence count data can be found in supplementary tables S1A-C).

The summary.single command was used to generate alpha diversity metrics by treatment and time. To prevent measuring spurious differences in alpha diversity resulting from variation in read depth between sequencing runs and across samples, libraries were rarified to 19,000 reads before calculating the number of observed OTUs (sobs), Shannon diversity, and Shannon evenness. Four samples were excluded due to library size below 19k^32^. The rarefaction and alpha diversity calculation process was iterated 5,000 times and resulting alpha diversity metrics were averaged across iterations.

Total hindgut bacteria were quantified using BactQuant^33^ qPCR primers in a SYBR-green assay on Bio-Rad CFX96 thermocyclers. To provide absolute quantification of 16S rDNA copy number and ensure inter-run comparability, in-run standard curves were included on each run. We created plasmid standards for each assay using either a 16S gene clone (using Invitrogen pCR^®^2.1-TOPO™ cloning vector (#K4500-40) and DH5α™ cells (#18265017) per manufacturer’s specifications), purified via plasmid mini-prep kit (Thermo Scientific #K0503). We determined DNA concentration (dsDNA/µl) via Implen NanoPhotometer P300, and the known mass of plasmid plus PCR insert was used to calculate 16S plasmid standard copies per µl. Standard curves were calculated from a 10-fold serial dilution of the plasmid standards included on each run.

### Microbiome size and structure

The 21 most abundant species designations by total read number accounted for 95.5% of all reads. Of these, four species had high read totals but both low prevalence across samples and most of their total read abundance concentrated in five or fewer samples. These four and the remaining 219 OTUs of lower abundance and prevalence were summed together as a measure of diversity abundance referred to herein as “Others.”

The 17 high abundance species included all 5 honey bee associated core members of the *Lactobacillus* genus previously known as Firm-5, as well as both core species of *Bombilactobacillus: B. mellis* and *B.mellifer*, *Bifidobacterium asteroides*,* Snodgrassella alvi*,* Frishella perrara*,* Apilactobacillus kunkeei*,* Gilliamella apis*,* Gilliamella apicola*, and *Commensalibacter melissae.* Another undescribed species in Gilliamella was assigned a placeholder name by BEExact, *Gilliamella sp. bxid9399.* The final two members were a *Gilliamella sp.* and *Lactobacillus sp.* neither of which could be classified at the level of species.

Read counts of these 17 species and Others were normalized to an estimate of bacterial cell number. First, the relative abundance of each species in each sample was calculated based on raw read count. Absolute species abundance was then calculated as the product of species relative abundance and 16S rRNA gene copies determined with BactQuant^33^. Absolute abundances were adjusted by dividing by the 16S rRNA copy number for each species, while Other was normalized with 4.2 gene copies, the mean 16S rRNA gene copy number averaged across all known bacteria^34^.

### Statistics

To perform parametric statistics, the sample abundance data were transformed using a centered log ratio (CLR) approach with CoDaPack^35^. This addresses the compositional constraints inherent in microbiome relative abundance analysis^36^.CLR-normalized data were used to investigate changes in the overall covariance structure of microbiota species using multivariate analysis of variance (MANOVA), examining tylosin and Age as independent variables and 18 dependent variables that represent the honey bee gut microbiome. Pillai’s Trace test statistic was used for all MANOVAs to account for deviations in normality and homogeneity of covariance. We used Wilcoxon tests on the qPCR-normalized estimated bacterial cell number at each time point by treatment to examine absolute abundance changes in individual species without regard to overall microbiota structure. We report the false discovery rate (FDR) and apply a Bonferroni correction to account for multiple comparisons. We performed principle component analysis (PCA) on the CLR scores for each hindgut sample to visualize the relationship of bacterial community composition with antibiotic disturbance and age-associated succession.

After BEExact classification, OTUs assigned the same species name were analyzed as a group for microbiome structure analyses. Within 13 of the 17 top abundance species, 1–3 unique OTUs made up the majority of reads. To determine whether tylosin treatment or age induced shifts in OTU representation within a species, we examined those species possessing multiple unique OTUs with > 10% of the total reads for that species and > 5% prevalence across samples. We performed Spearman’s correlations on the absolute abundance of each OTU and performed Fisher’s exact test on counts of OTU occurrence by sample. We used Wilcoxon tests on absolute abundances to examine differences in OTU proportions across samples, within genera and select species.

Microbe-microbe interactions may be important drivers of microbiome assembly, with cooperative or competitive interactions determining which strains associate in the mature microbiome. Our 16S amplicon data cannot resolve true strain-level differences, but to test for associations between OTUs within named species, we assessed pairwise co-occurrence among the high-abundance OTUs using null model simulations. For each pair, we calculated the observed Jaccard similarity based on shared and total presences across samples. To determine if the observed co-occurrence was significant, we compared it to a null distribution of Jaccard values generated from 1,000 randomized datasets. These randomizations preserved sample richness and taxon prevalence while shuffling specific pairwise relationships. A two-tailed test was used to calculate p-values, testing for significant aggregation (positive association) or segregation (negative association). P-values were adjusted for multiple testing using the Benjamini-Hochberg FDR method, and taxa pairs were classified as aggregated, segregated, or random.

## Results

### Overall microbiome abundance dynamics

**Fig. 1:**
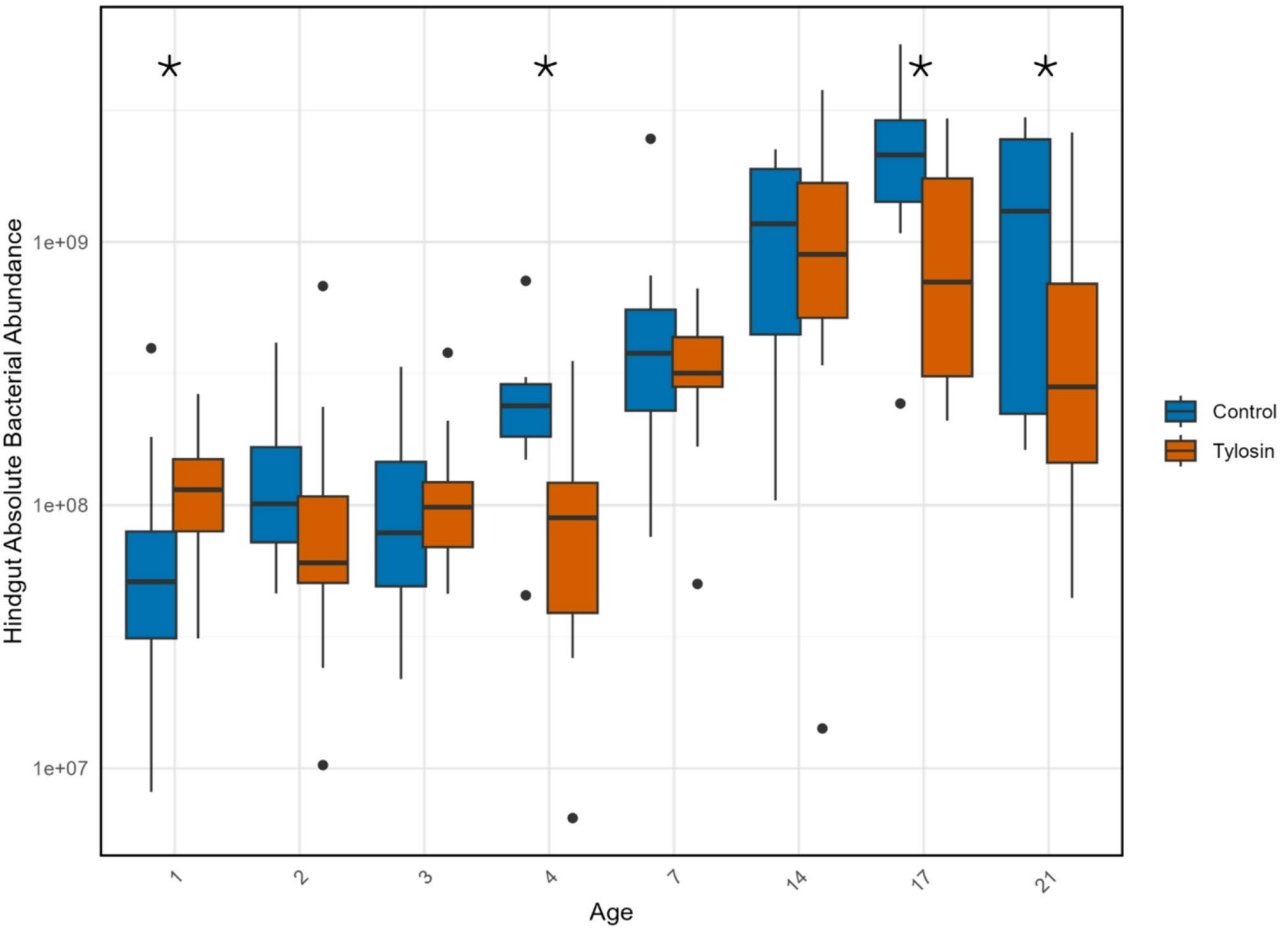
Absolute bacterial abundance as estimated by bactquant, after species-specific adjustments for 16S copy number; Age is time in days after bees were placed into colonies; * = P value < 0.01.

Microbiome size varied across timepoints (ANOVA), with tylosin significantly affecting absolute abundance by day (Wilcoxon tests). The bacterial load in both control and treated bee hindguts increased consistently over time to a peak at days 14–17, then decreased by day 21 (Fig. 1). In tylosin-treated bees, total abundance increased more slowly and peaked at a lower median value.

**Fig. 2:**
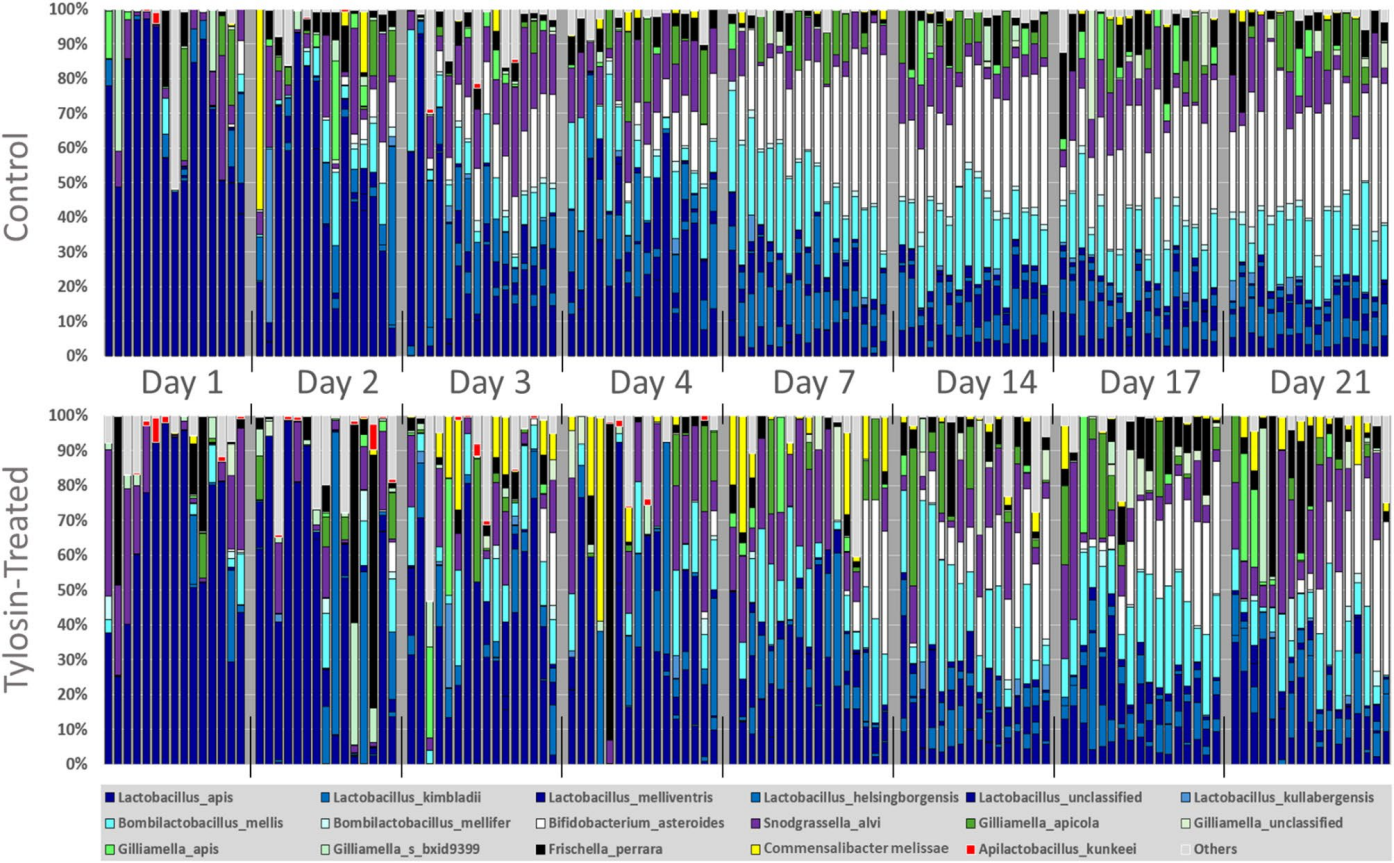
Hindgut relative bacterial abundance by species.

We found that bees at one day of adulthood possessed a total hindgut bacterial abundance with a median of approximately 2 × 10^7 bacteria. Hindgut bacterial abundance in one-day-old workers was significantly higher in tylosin-treated bees (Fig. 1; ANOVA *p* < 0.01), about twice as high on average. In addition to greater total bacterial abundance, both proportional and absolute abundance of the group Others (a proxy of diversity abundance, comprised of the sum of rare core taxa, all noncore taxa, and contaminant taxa) was higher in treated bees, and the count of unique OTUs was significantly higher (Fig. 2; Wilcoxon Rank Sum, *p* = 0.037; Table S4).

For each day after the third, median bacterial abundance was lower in tylosin-treated hindguts than in controls, though these differences were only significant only on day 4, with trends toward significance on days 17 and 21. Median total abundance reached a peak in control bees on day 17, but the peak for tylosin treated bees came earlier on day 14. After these ages, both groups saw a decline in median absolute bacterial abundance (see Fig. 1).

### Species abundance dynamics

In addition to changes in overall community size, microbiome structure also shifted with age and treatment. We measured relative and absolute abundance of the top 17 species and summed Others group using MANOVA and Wilcoxon rank sum analyses.

Two-way MANOVA revealed that community ratio abundance structure differed by both time (Pillai’s Trace, *p* < 0.0001) and antibiotic treatment (*p* < 0.0001), with a significant interaction effect (*p* < 0.0001). Following FDR correction, 8 of 18 analyzed categories differed by tylosin treatment, 10 differed by age, and 4 by the interaction of antibiotic treatment and age (For MANOVA details, see Supplementary Tables S2A-C).

PCA on CLR-adjusted relative abundance scores showed little separation between tylosin treated and control microbiomes, but clearer differences between the youngest and older bee guts (Supplementary table S3).

Ten of the 17 species, showed significant absolute abundance differences by treatment (Wilcoxon), while *L. melliventris*,* Lactobacillus sp. unclassified*,* F. perrara*, and all *Gilliamella* species were unchanged by treatment (Wilcoxon results and plots of each species’ abundance over time by treatment can be found in Supplementary Table S4).

### Core species strongly affected by tylosin

*B. asteroides* and *B. mellifer* exhibited the most drastic changes in response to treatment. The MANOVA model found a significant effect of age, tylosin, and the interaction factor for each. In controls, relative abundance increased steadily throughout the study, while in treated bees it remained flat until day 7, then rose gradually. Both species established in controls by days 3–4 and comprised up to one-third of total abundance by days 14–21 (see Fig. 2, abundance plots in table S4B). Treated bees showed a lower prevalence of both species, with significantly more samples showing failure to establish compared with controls (Fishers Exact test, *p* < 0.001, Table S5A-D).

### Moderately affected or unaffected core members

*L. apis* was the only species found in every bee. Its relative abundance in both treated and control bees was very high on day 1, with most hindguts dominated 65–75% by this species. This proportional abundance then declined over the first few days to around 10% and remained stable until the end of the experiment. *L. apis* absolute abundance remained stable across age except for a peak at day 17 for controls (Kruskall-Wallis with Steel-Dwass *p* > 0.05). Tylosin treated bees failed to show the same high day 17 absolute abundance but otherwise mostly resembled controls in *L. apis* abundance.

Other *Lactobacillus* and *Bombilactobacillus* species showed similar or slightly reduced abundance in treated bees. In general these species established after day 4 and frequently exhibited slightly lower abundances in tylosin treated bees at most time points (See detailed abundance plots in table S4B).

Core species little affected by either age or tylosin exposure included *S. alvi*,* G. apicola*, and *G. apis.* These ileum-colonizing species showed no significant effects for treatment or age (MANOVA) (Table S2-4). Wilcoxon rank sum tests showed that *G. apicola* and *G. apis* also exhibited flat absolute abundance over time (Table 4). *S. alvi* abundance did increase in control bees along with the peak in total bacterial abundance, but this increase was not matched by treated bees (See detailed abundance plots in table S4B).

### Non-core or opportunistic species trends

*F. perrara* relative abundance showed no relationship with age or tylosin in the MANOVA model and its absolute abundance did not differ by tylosin treatment. This species was found in most bees and co-occurred with *G. apis* but was negatively associated with *G. apicola* (Spearmans correlations, null model analysis, and Fisher’s exact tests, Table S5).

*C. melissae* absolute abundance and prevalence were higher in treated bees overall but when examined by age only showed significantly greater absolute abundance on day 3. MANOVA revealed relative abundance from days 1 to 3 increased for tylosin-treated bees but decreased for controls, remaining stable in both groups after this initial period.

*A. kunkeei* was prevalent at low absolute abundances in younger (day 1–4) bees, especially when treated with tylosin. Both treated and control bees showed a decline in *A. kunkeei’s* absolute and relative abundance over time as the rest of the microbiome expanded, with MANOVA identifying a significant effect of age on this pattern. MANOVA also revealed a significant effect of tylosin, with treated bees consistently exhibiting higher relative abundances.

The ‘Others’ group was significantly higher on day 1 in treated bees and remained modestly elevated throughout most of the experiment, approaching the same proportion as in control bees towards the end.

### Alpha diversity

**Fig. 3:**
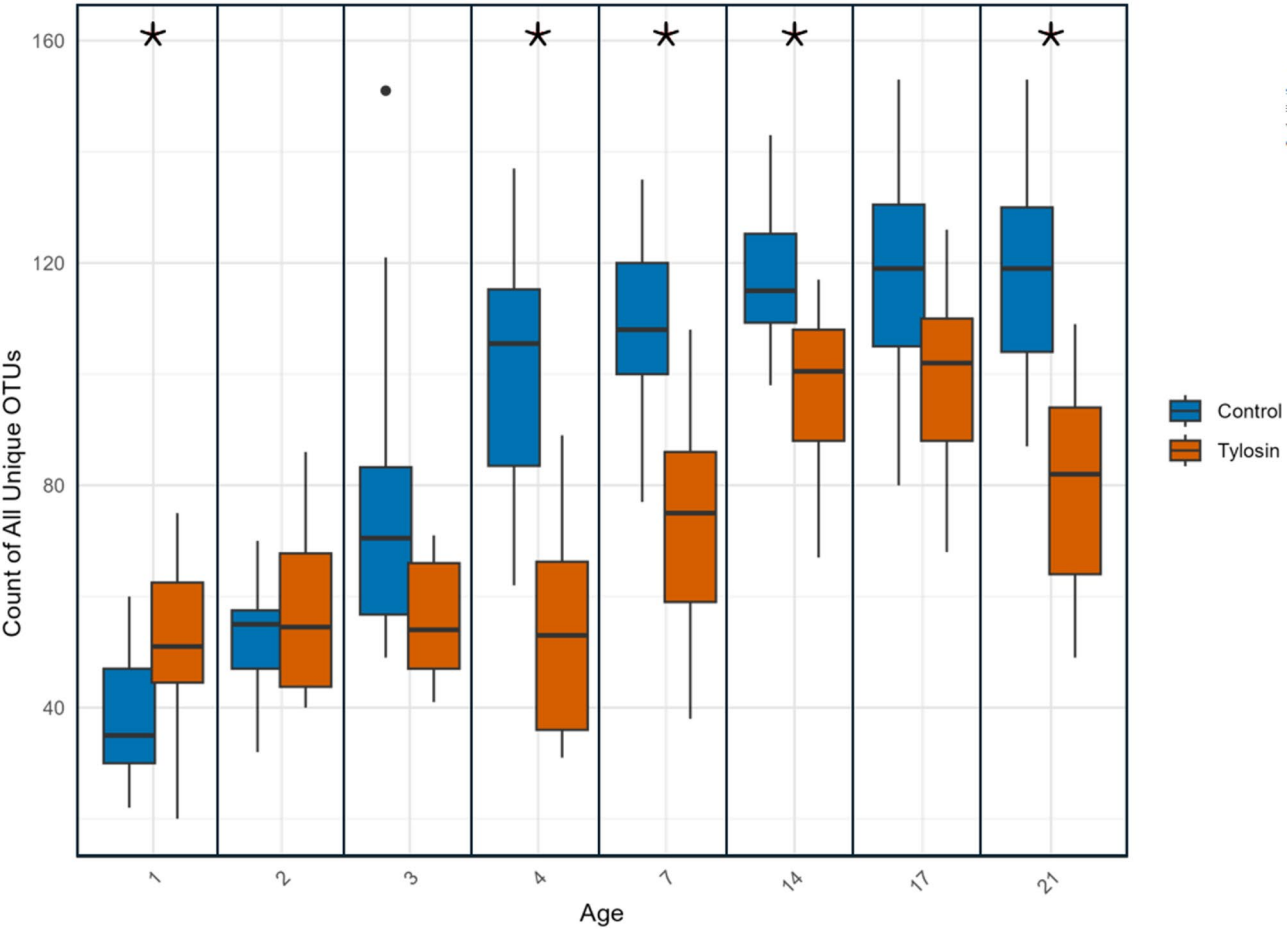
Unique OTU count, Age is time in days after bees were placed into colonies; * = P value < 0.01.

To test the hypothesis that antibiotic application disturbs acquisition of diverse elements of the gut microbiome, we examined the Shannon diversity, Shannon evenness, observed species number, and effective species number on rarefied read counts. In all bees, all four metrics increased with age. Tylosin treatment reduced or delayed this increase. Though Observed species number was not significantly different between treated and control bees, Shannon diversity and evenness were significantly lower for most days after the 3rd, and Effective species number was correspondingly significantly reduced. Treated microbiomes were less diverse and less even, but the median difference was greatest for days 2–7, becoming closer to control values towards the time of typical onset of foraging (Fig. 3, Table S6).

### Diversity within species: unique OTUs

The total number of unique OTUs counted in the guts of all tylosin treated bees was lower than that of controls (Fig. 2). On day 1, unique OTU count was higher in treated bees, but increased more slowly than controls, remaining lower or indistinguishable from controls for every day after the first. These differences were largely dependent on the differences in diversity in the genera *Lactobacillus*, which contained 50% of all OTUs in the dataset, *Bombilactobacillus* (14%), and *Bifidobacteria* (5%) (See Tables S6A-D). Diversity, as represented by unique OTU counts in these genera, remained lower in treated bees as that of control bees climbed.

Across all detected bacterial species as classified by BEExact, the number of unique OTUs per species numbered in the tens to hundreds. All unique OTUs assigned to each species name were summed together for analyses of absolute and relative abundance and alpha diversity assessments, however certain OTUs made up the majority of detections for many species. For 15 of the 17 top species, only one to three OTUs exceeded 10% of the abundance total for that species, and the sums of these few OTUs covered 80–99% of abundance for each of those species (Table S6C). To determine whether age or antibiotic treatment had effects on the OTU composition within named species, we examined patterns of occurrence of these highest abundance OTUs.

For true strain-level analysis, sequencing only 16S rRNA cannot resolve the full diversity within species in the hindgut, but we found that core species were composed primarily of 1–3 high-abundance OTUs plus a tail of many more of very low abundance. Examining these high-abundance OTUs revealed a consistent pattern where OTUs belonging to *L. apis*,* L. helsingborgensis*,* L. melliventris*,* L. kimbladii*,* B. asteroides*,* B. mellis*,* F. perrara*, and *S. alvi* show a clear pattern of segregation at the individual bee level. One OTU representative of the species dominates each individual gut with strong negative correlation or total exclusion of other OTUs in the same species (Spearman’s rho and Fisher’s Exact Tests, Table S6C).

High abundance OTUs of *G. apicola*,* G. apis*, and *G. unclassified* extend this pattern of exclusive OTU dominance within species to exclusion between species. Each high-abundance OTU of these species was negatively correlated with other OTUs of the same species, but also with each OTU of the others (Fisher’s Exact Tests, Table S6C-D). Only 6 of the 240 samples contained any combination of a high abundance OTU of *G. apicola*,* G. apis*, or *G. unclassified* alongside another high abundance OTU of any of these species. Many other lower abundance *Gilliamella* OTUs (those representing < 10% of species abundance) co-occurred frequently with these and with each other, but never represented a substantial part of the bacterial abundance for any sample. Sample sizes limit the ability to discern whether these OTUs show differences in prevalence or abundance between treatments or across ages. *G. apis* OTU0026 prevalence and absolute abundance were lower in tylosin treated than control bees, but neither of these differences were significant after FDR correction (Table S5A).

Within the genus *Lactobacillus* (formerly known as Firm-5), null model analysis showed some OTUs to interact with OTUs of other species (Table S6D). *L. kimbladii* OTU0011 had a negative relationship with *L. kullabergensis* OTU0023, but a positive relationship with *L. helsingborgensis* OTU0004. *L. kimbladii* OTU0016 showed the opposite, with a positive relationship to *L. helsingborgensis* and no relationship with *L. kullabergensis* OTUs. The least abundant *L. kimbladii* OTU0027 had a slight positive relationship with *L. kullabergensis* OTU0023. Though species-level absolute abundances for these species showed some significant differences with treatment, sample size limitations due to low prevalence limit the ability to distinguish how these OTUs individually were influenced by tylosin. (Fisher’s Exact Tests, null model simulation, Tables S5B, S6D).

Despite absolute and relative abundance differences of many species overall, the prevalence and abundance of the OTUs within most core species did not vary with age or tylosin exposure. Two species showed effects of tylosin on OTU prevalence: *B. asteroides* and *S. alvi*. As expected from the effects on the species overall, *B. asteroides* OTU0005 was significantly reduced in absolute abundance and significantly more likely to be absent in treated bees, but *B. asteroides* OTU0024 showed no differences in either prevalence or abundance. For *S. alvi*, the more abundant OTU0002 showed no differences by treatment in prevalence or absolute abundance, but OTU0014 was significantly greater in treated bees by both metrics (Table S5A).

For other core species, we found no differences in OTU representation by age or treatment.

## Discussion

The mature honey bee gut microbiota presents a highly consistent and predictable structure, providing a model to study microbiome colonization and succession dynamics, especially in reaction to disturbances. During worker pupation, the larval microbiome is shed, and the adult microbiome then establishes over the first week following emergence. In this contribution, we exposed newly emerged worker bees to antibiotic (tylosin) treatment and recorded the age-specific change in gut microbiome size and structure. According to multiple metrics, tylosin treatment of NEWs resulted in gut dysbiosis, a state that persisted until the time of typical foraging onset (21 days). Combined with previous microbiome results sampling worker bees of unknown age during a mild winter^10^,antibiotic induced gut dysbiosis produced by colony level antibiotic application likely endures for the life span of the exposed worker.

This paper is the first comprehensive look at early natural succession in the worker gut. A previous comparison between microbiomes at ages 3 and 7 days showed a strong shift in the type of L*actobacillus* firm5 and an increase in firm4 from 3 to 7 days consistent with this work, but this study used observation colonies so the hive and social context were somewhat compromised^37^. Other previous studies of succession and antibiotic influences used lower taxonomic specificity, a coarser sampling over time, or reared bees in artificial lab conditions^5,6,9,38,39^. Here we performed a colony-level experiment with a high sampling rate. The context of social and colony materials exposure was natural, as was the diet of beebread and honey, and the ability to exit the colony for defecation and foraging, all important determinants for microbiome exposure and succession^13^. Some similar work has an advantage over our study in taxonomic discrimination because 16S rRNA-based methods cannot resolve true strain-level differences. Nevertheless, using 16S rRNA amplicons and classification with BEExact, we were able to detect high-abundance and prevalence variants within species that are unlikely to represent sequencing errors. This experiment provides a uniquely detailed picture of species succession and overall abundance patterns in the worker gut.

Hindgut bacterial abundance of one-day-old workers was significantly higher in tylosin-treated bees as was unique OTU abundance, suggesting that the antibiotic environment alters the selective nature of the pristine adult gut environment of NEWs. This greater abundance of diverse bacteria may reflect lowered barriers to initial colonization as core microbiome members with negative influence on noncore members are slower to establish. Antibiotics also induce host oxidative stress, and may alter the gut environment to make host factors less able to suppress opportunist species or encourage growth of core microbiome members^40^.

Total bacterial abundance increased for both treated and control bees until days 14–17 and then declined. As bees transition from young and middle-age bee tasks such as nursing and guarding to foraging, total bacterial abundance and relative proportion of several species decreases^11,21,41^. Forager nutrition shifts away from pollen to almost exclusively nectar, leading to decreases in abundance of pollen-fermenting species. We did not monitor behavioral changes among marked bees, but the peak microbial abundance came earlier in treated bees. Tylosin exposure at different ages and stages of development can alter patterns of behavioral development in workers^42^. If the drop we measure in bacterial abundance following the peak reflects the transition from middle-age bee tasks to foraging and its associated metabolic and nutritional changes, the earlier peak in tylosin treated bees may reflect an earlier transition to foraging behaviors. Species-specific effects on taxa associated with behavioral and developmental changes were also consistent with this hypothesis^13^.

*Lactobacillus apis* is a major early colonizer of the hindgut, quickly followed by the other core microbiome members. We found that tylosin-treated bees in the first day post adult emergence established an abundance of *L. apis* similar to control bees. *L. apis* relative abundance in treated bees was slightly higher across time points, despite little difference in absolute abundance. This may be because total abundance of other taxa was reduced by tylosin while L. apis levels remained relatively unchanged. Other species failed to establish at their usual rate, creating a smaller, less even, and less diverse community. These differences were at their greatest by about the 4th day, and afterwards, treated microbiomes became more similar to controls. Other *Lactobacillus* species began to establish soon after adult emergence. These species reacted differently to treatment, with *L. kimbladii* proportional abundance higher under tylosin treatment but *L. helsingborgensis* abundance lower. *L. kullabergensis* and and *L. melliventris* did not respond to treatment significantly, in concordance with previous results^6^. *Lactobacillus* species partition their shared hindgut niche by utilizing different pollen-derived carbohydrate sources^43^. The unequal effects of antibiotic treatment on these species likely influences the availability of specific pollen-derived nutrients and their secondary metabolites.

Among all core species, *B. asteroides* and *B. mellifer* were most strongly suppressed by tylosin. These species normally establish early and become dominant by two weeks of age, but their prevalence and abundance were significantly reduced or delayed in treated bees. *B. asteroides* failed to establish by days 3 and 4 in many tylosin-treated bees despite making up around 10% of the relative abundance of hindgut bacteria on those days in controls. This species is responsible for a significant fraction of gut metabolic output in healthy bees and may participate in cross feeding interactions with other species^44^. *Bifidobacteria* show protective effects against pathogen infection in bees and other animals^45^. In addition, *B. asteroides* has many reported relationships to the age and behavior of bees, including the levels of host developmental hormones^44^gene expression and GABA concentration in the brain^22^and foraging behavior^46^. The dramatic decrease or failure to establish of this species in treated bees could cause developmental changes in workers. Across multiple studies, the strongest abundance correlation among core hindgut species occurs between *Bifidobacterium* and *Bombilactobacillus* species^13^. *B. mellifer* also largely failed to establish in treated bees. Despite close relation to *B. mellifer*, *B. mellis* proportional abundance remained about the same with treatment, revealing differences in antibiotic resistance between these species. Like *Bifidobacteria*,* Bombilactobacillus* species have been shown to influence brain development and specifically to modulate olfactory functions, suggesting that disturbances to the species abundance relationships within this genus may have behavioral effects^4,46,47^. Low *Bifidobacterium* and *Bombilactobacillus* abundance and a peak in total bacterial abundance followed by decrease are all associated with foraging onset, suggesting potential developmental or behavioral effects of tylosin on the treated workers. Although recent literature^41^supports such an interpretation, other mechanisms could also explain these differences, such as changes to host gut physiology. Whether tylosin-induced dysbiosis can influence age at foraging onset should be explored in future research.

More *C. melissae* (a queen gut bacterium) colonized tylosin treated bees on the third and fourth days. *Commensalibacter* is gram-negative, and *C. melissae* is a dominant core microbiome member in queen guts, correlated with markers of queen health and fecundity^12^. Its function in the worker microbiome is unknown but its presence is more sporadic, previously reported as increasing in prevalence as workers age^14^. We found that in treated bees, absolute abundances of *C. melissae* decreased over time but relative abundance remained high in many bees, even at 21 days of age. The bloom of this taxon in the early worker gut and its continued presence as part of the tylosin-treated gut may be an example of metabolic niche opportunism, as it seemed to increase in relative abundance as groups associated with fermentation of complex sugars decreased^48^. *C. melissae* has a complete TCA cycle and can use other bacteria’s excreted metabolites as fuel, so metabolite balance differences in antibiotic-induced dysbiosis may explain its appearance in treated bees. Higher relative abundance of *C. melissae* was also associated with decreased proportions of *B. mellifer* and *B. asteroides* and these species have each been associated with degradation of ω-hydroxy acids in the pollen coat^44,49^. *C. melissae* colonization of the worker gut may depend on availability of these resources, appearing in treated bees depleted in *B. mellifer* and *B. asteroides* populations.

Ileum colonizing species *S. alvi*,* F. perarra* and all four *Gilliamella* species were not strongly affected by tylosin treatment and showed little change in either absolute or relative abundance by worker age. *F. perarra* and *Gilliamella* species and OTUs showed significant associations, both positive and negative. Other work has shown conflicting relationships between these species, illustrating the dynamic microbial interactions within the ileum niche^43,50,51^. The positive correlation between *F. perrara* and *G. apis* could simply reflect increased resource availability in their shared niche, as both species colonize the anterior portion of the gut at or near the pylorus and possess genes for urea utilization. *G. apicola* did not show the same association, but tends to colonize further along the ileal tract^52^. *G. apis* is a late-establishing microbiome member and may depend on temporal changes in host physiology or on the establishment of specific partner bacteria for colonization^13^. *S. alvi* strains vary in type VI secretion systems, and interact intimately with the host and *Gilliamella* strains in the essential oxygen-depleting ileum biofilm^50^. Tylosin treatment effects on the availability of compatible *S. alvi* strains could be important for both host-microbe and microbe-microbe interactions that drive microbiome strain assembly, and future work should investigate the role of strain identity on community assembly in the honey bee gut. Our OTU frequency results are consistent with a shift in *S. alvi* strain populations to favor a more antibiotic-resistant strain within the treated colony, though in our data this cannot be distinguished from colony-level differences in strain representation, unrelated to treatment. These relationships between the species and strains in the ileum with time and treatment require greater sampling effort and higher resolution sequencing to confirm conclusively, but suggest that strain availability may be an important driver for microbiome assembly and that antibiotic effects could negatively impact this availability.

The Others group, representing the diversity abundance of non-core species, showed higher relative abundance throughout the early life of treated workers, but absolute abundance was only significantly higher on the first day. This implies that the higher relative abundance was the result of an overall smaller microbiome size in treated bees, with core taxa abundances decreased. This pattern may reflect delayed colonization by core taxa, enabling early overrepresentation of rare or non-core microbes.

Many core species showed very little change in either relative or absolute abundance, demonstrating a lack of sensitivity to tylosin in the conditions of our study. As tylosin primarily targets gram positive bacteria this is partly expected, but we note differences from previous reported tylosin sensitivity among the core microbiome. *S. alvi* was essentially stable in our results, showing no significant changes in abundance with treatment or time despite previous work showing tylosin-induced reduction in abundance and strain diversity^6^. Antibiotic resistance genes providing resistance to tetracycline have previously been identified in many of the core microbiome members, including *C. melissae* and *S. alvi*^27^. Genes putatively conferring tylosin resistance have been identified in gram positive bacteria residing in honey^53^. Macrolide resistance genes have been recorded in some core microbiome bacteria, though tylosin treatment did not significantly increase their frequency in treated bee guts^54^.The lack of tylosin sensitivity for *S. alvi* in our experiment could be due to such resistance genes, though we did not test for these.

At the level of unique OTUs, we found a pattern of negative correlation or total exclusion of OTUs classified to the same species by BEExact. This pattern is consistent with previous research on strain-level diversity between bees and may represent the effects of individual OTUs being first to establish in the gut, interactions with host genetics, or competitive interactions between strains^21,49,55^. For two species, we found OTU-level differences in prevalence and abundance by treatment. Among OTUs classified as *B. asteroides* and *S. alvi*, specific OTUs were more prevalent overall in treated bees. This pattern is consistent with tylosin treatment selecting for more resistant strains within the colony as has been demonstrated for oxytetracycline treatment^56^.

Within the other core species, we found no differences in OTU representation by age or treatment. Strains of these species may have little difference in tylosin sensitivity, or else tylosin effects may be less important than founder effects or host influence in the determination of what strain will dominate in the gut of a given bee. A recent study used shotgun metagenomics to examine strain turnover between nurse and forager bees and found that strain-level composition of the worker microbiome does change with task, but this work used pooled samples of multiple guts and cannot distinguish whether this turnover is due to acquisition of new strains later in life or changes in abundance of strains acquired early^21^. Although 16S rRNA results lack the resolution for true strain-level analysis, the lack of age impacts on prevalence of most OTUs in our results is consistent with strain-level segregation to within individual bees, suggesting that factors such as early colonizer identity and host genetics determine the lifetime strain composition of core species within a given worker.

### Limitations

Because bees in this experiment emerged in an incubator on frames from a common pool of colonies being marked and introduced to the treatment colony, it is possible that founder strains were acquired prior to colony introduction and tylosin exposure^15^. If founder effects and competition dominate early strain acquisition in newly emerged bees, this may limit the current study’s ability to distinguish whether antibiotic treatment biases strain acquisition and prevalence. In addition, the mutual exclusivity of same-species OTUs within individual bee guts means that any one OTU within a species may have a much lower prevalence than that species overall across the dataset, making effective sample sizes smaller and statistical comparison across treatment groups challenging. To conclusively show how tylosin treatment or the effects of age can result in changes to the prevalence and abundance of OTUs would require a greater sampling effort than that in this study. Sequences in this study were obtained in two separate MiSeq runs which were combined for downstream analysis. Though alpha diversity did not differ overall between runs and ordination plots did not cluster by run, it is possible that sequencing batch effects could be responsible for spurious differences between early and late age microbiomes. Finally, the stark differences in *Bifidobacterium* and *Bombilactobacillus* abundance and other major effects between treatments are large effect sizes and consistent with other work and expectations for tylosin effects on the gut microbiome, however the use of two study colonies risks confounding colony differences with treatment effects for observations of smaller effect sizes.

## Conclusions

Here we establish a comprehensive baseline for how the newly emerged honey bee worker gut microbiome is assembled in early life and changes through to the typical time of the onset of foraging. We then show how a common beekeeping antibiotic application interferes with this normal assembly. Tylosin-exposed newly emerged adults develop a putatively dysbiotic microbiome deficient in certain core members and lower in total bacterial abundance. Initial microbiome community size and structure on day 1 was similar but differences became increasingly apparent as bees aged. Many treated bees continued to show high relative abundance of noncore species such as *C. melissae* and little abundance of essential core members like *B. asteroides* even at day 21 (Table S4B, Fig. 2). This dysbiotic state is persistent at least 21 days post emergence from the pupa and likely longer, and the specific absences of core microbiome members suggest effects on worker behavior and development.

Coexistence in the gut of one bee was very uncommon for the most abundant OTUs for each species. This result is consistent with the hypotheses that host genetic influences or initial colonization by a given strain and subsequent competitive exclusion determine the strain makeup of a given gut microbiome. In either case, this suggests that selective depletion of certain strains through mechanisms like antibiotic treatment could contribute to forming dysbiotic microbiomes deficient in strain membership or functional capacities. Our measurement of higher prevalence of *S. alvi* OTU0014 in treated bees could be consistent with such an effect but more conclusive evidence should be sought in future studies. Our results provide another example in a growing set of studies that show how use of technically non-toxic chemicals and antibiotics may nonetheless negatively impact bees through unintended effects on essential microbial associates.

## Electronic supplementary material

Below is the link to the electronic supplementary material.

Supplementary Material 1

## Data Availability

The raw sequence read datasets generated for this study are deposited in GenBank, SequenceRead Archive BioProject PRJNA1241321.
